# Wireless Sensor Network Combined with Cloud Computing for Air Quality Monitoring

**DOI:** 10.3390/s19030691

**Published:** 2019-02-08

**Authors:** Patricia Arroyo, José Luis Herrero, José Ignacio Suárez, Jesús Lozano

**Affiliations:** Industrial Engineering School, University of Extremadura, 06071 Badajoz, Spain; parroyoz@unex.es (P.A.); jherrero@unex.es (J.L.H.); jmarcelo@unex.es (J.I.S.)

**Keywords:** chemical sensors, wireless sensor network, cloud computing, air quality

## Abstract

Low-cost air pollution wireless sensors are emerging in densely distributed networks that provide more spatial resolution than typical traditional systems for monitoring ambient air quality. This paper presents an air quality measurement system that is composed of a distributed sensor network connected to a cloud system forming a wireless sensor network (WSN). Sensor nodes are based on low-power ZigBee motes, and transmit field measurement data to the cloud through a gateway. An optimized cloud computing system has been implemented to store, monitor, process, and visualize the data received from the sensor network. Data processing and analysis is performed in the cloud by applying artificial intelligence techniques to optimize the detection of compounds and contaminants. This proposed system is a low-cost, low-size, and low-power consumption method that can greatly enhance the efficiency of air quality measurements, since a great number of nodes could be deployed and provide relevant information for air quality distribution in different areas. Finally, a laboratory case study demonstrates the applicability of the proposed system for the detection of some common volatile organic compounds, including: benzene, toluene, ethylbenzene, and xylene. Principal component analysis, a multilayer perceptron with backpropagation learning algorithm, and support vector machine have been applied for data processing. The results obtained suggest good performance in discriminating and quantifying the concentration of the volatile organic compounds.

## 1. Introduction

Environmental pollution is a major problem both in developed and emerging countries. Increasing air pollution not only affects global warming, but also directly affects human health. The International Agency for Research on Cancer (IARC) has evaluated and verified the carcinogenicity of external air pollution [1]. In 2015, 16% of deaths worldwide were caused by pollution-related diseases [2]. Besides, if a control is not implemented, these numbers will tend to increase. Natural and anthropogenic (traffic and industry) emissions are the major sources of air pollution. Volatile organic compounds (VOCs) are among the main pollutants. They are organic chemical compounds with high vapor pressure under normal conditions, so they vaporize easily in the atmosphere. Specifically, high interest exists in four chemical components found in petroleum products. These components, commonly called BTEX, are benzene, toluene, ethylbenzene, and xylene. BTEX pollutes the environment mainly near oil and natural gas refineries, gas stations, and other areas with storage tanks. These compounds are a point of interest in research because acute exposures to high levels have been associated with skin irritation, central nervous system depression, mental disorders, and effects on the respiratory system. Specifically, studies show that the short exposure of benzene in the human body produces drowsiness, headaches, and dizziness. However, prolonged exposures are related to aplastic anemia, acute leukemia, and bone marrow abnormalities [3,4,5]. According to a European Union (EU) directive effective on December 2000, concentrations of benzene in ambient air should not exceed five µg/m^3^ as an operating annual average, with a long-term target of less than one µg/m^3^ [6]. In this context, new sensor technologies could provide a potential new method for the short-term monitoring of BTEX that may be relevant to health.

Therefore, the monitoring of pollutants and VOCs is extremely important. The data acquired by the stations with traditional measurement instruments can be used to construct maps and pollution models that provide environmental situation information and authorized prediction. However, limitations in spatiotemporal resolution are a common condition in these systems. These limitations result in problems for conventional air pollution monitoring systems, such as the non-scalability of the system or the limited availability of data on personal exposure. Due to this, different small, portable, and/or low-consumption devices have emerged. Although these devices are less accurate than reference systems, their use increases the spatial density of the measurements. In other words, they are not intended to replace reference instruments, especially for enforcement purposes, but rather are a complementary source of information on air quality. In this way, atmospheric models and predictions can be validated at high temporal and spatial resolution [7]. Some of these devices were summarized by author McKercher [8]. Among them is the Smart Citizen system, which has emerged [9] as one of the most innovative approaches. However, despite using MOS sensors for measuring gases (with high cross-sensitivity), this proposal does not include a machine learning system to allow a final prediction. On the other hand, Sens-it [10], which was developed by Unitec, measures a large amount of pollutants, but is designed to be integrated into an existing device. Magnasci SRL has developed devices to monitor pollution. Among the most significant, it is worth mentioning the URad A3 device [11], which requires a station for its installation, since it is not a wireless device. Otherwise, URad Industrial [12] itself is a wireless device, but it is limited to the measurement of four compounds, since it contains specific sensors for each pollutant to be measured.

Wireless sensor networks (WSN) [13] have a great role in this field, enabling the mitigation of many of the problems in traditional monitoring systems. They provide a continuous and distributed operation, which is very important in this field, achieving the necessary increase in the spatial density of the measurements. Furthermore, in this way, contaminant measurement systems with low-energy consumption, complexity, and cost are achieved. There are several communication technologies available to be used in WSNs, and their choice will depend on the environment and the application concerned. For example, if a high data rate and high range are necessary, it is recommended to use IEEE 802.11 protocol. In addition, there are protocols created for specific applications, such as ISA100 and WirelessHART, which are used for industrial control and process-monitoring applications. Low-power wide-area network (LPWAN) technologies are a type of wide-area wireless telecommunications network that is designed to enable long-range communications at a low bit rate between connected objects. Although it does not allow a mesh type configuration, losing network reliability, it is a solid choice for implementing low-power, low-cost, and long-distance WSNs. LPWANs include specifications such as NB-IoT (Narrowband-IoT), Sigfox, or LoRaWAN (Low Power Wide Area Network) [14]. Moreover, for low-consumption applications that do not require high scope, other communication technologies are used, such as ZigBee, Wibree, and Ultra Wide Band. In particular, ZigBee is mainly oriented for low-cost applications with batteries where a low data rate is required. Since in many applications the communication module spends much time in power-saving mode, the devices are able to remain operational for long periods of time before their batteries need to be replaced. This protocol has been used in many fields, including environmental monitoring and the detection of compounds [15,16,17].

In recent years, two other emerging technologies have also been applied in this field: Cloud Computing and the Internet of Things (IoT). First, cloud computing is a term that is used to describe servers from the Internet that are responsible for responding to requests at any time. In addition, it allows access to information or service through an Internet connection from any mobile or fixed device located anywhere. This technology offers important advantages such as virtually unlimited storage and high processing capacity. Otherwise, the IoT technology is the interconnection of a network of self-configuring objects with the Internet. These two technologies are complementary, since the limitations of one can be covered by the other. In other words, IoT can benefit from the unlimited capacity, storage, and communication offered by Cloud technology while, from the other side, Cloud can reach out to the "real world" through the IoT [18,19]. Although it is a new infrastructure, this type of application has already emerged in different areas such as health [20,21], smart cities [22,23], or environmental monitoring [24,25,26]. In addition, following the merger of Cloud computing and IoT, some platforms that offer services to store and/or process information from the IoT in the cloud have emerged [27]. Among them are open source projects, such as OpenIoT [28], or commercial clouds offered by service providers such as Xively [29], ThingSpeak [30], CloudPlugs [31], DeviceCloud [32], Thinkingthings [33], SensorCloud [34], AWS IoT [35], or Google Cloud IoT [36]. However, in this paper, we have chosen to use our own cloud, since it gives us the advantage of customizing it according to our application requirements, including machine learning functions, while at the same time remaining independent from private alternatives.

Among all of the pollutants present in the atmosphere, VOCs are those with the most limited set of observational data. For this reason, it may be the most benefited field by low-cost sensors. Among these types of sensors, the resistive, electrochemical, dispersive infrared radiation absorption (NDIR) and photo ionization detector (PID) sensors stand out [37]. However, due to its low price, size, and power consumption, and its ability to detect a large number of gaseous compounds, the resistive metal oxide (MOS) sensors are the most commonly used. This kind of sensor offers significant variability, but has been shown to be reasonable in relation to atmospheric variability [38]. Therefore, its use, together with a selective and sensitive traditional technique, can improve the information regarding the presence of VOCs. In addition, they provide continuous measurements, and have fast enough responses to identify real-time contamination events. Finally, there are studies that show non-linear responses of MOS sensors in the presence of VOC [39,40]. However, their response becomes linear at concentrations below 100 ppb [41], which is considerably higher than the usual value in the atmosphere.

While in this case the system has been optimized for the discrimination of BTEX compounds, it could be used for the discrimination of other compounds thanks to the use of resistive sensors, which react to a high number of them. Consequently, the use of pattern recognition and artificial intelligence techniques is necessary, as they allow the system to be trained for any application. The main techniques that are used in this field have been reported in recent literature [42,43,44,45].

The system is detailed in the different sections of the paper. First, the design of the sensor nodes, wireless communication, and its configuration are described, along with the data processing and artificial intelligence techniques that are used, and the data cloud system. A laboratory demonstration of the operation of the system is then carried out. Then, the BTEX measurement and discrimination experiments at different concentrations carried out in the laboratory are presented. Finally, the results and conclusions obtained are discussed.

## 2. Materials and Methods

In this section, a cloud-based system to monitor air quality and detect specific compounds that are present in the environment is presented. To this end, a cloud-connected wireless network of gas sensors has been created. The evolution of the device up to the current one (introduced in this paper) has been already described in previous works [46,47,48].

**Figure 1:**
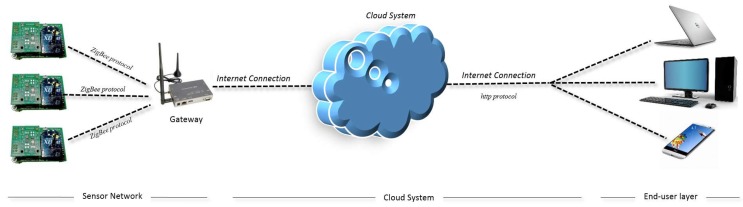
Cloud sensor network scheme.

The cloud sensor network is presented in Figure 1, which is composed by three different sections: a sensor network, a cloud system, and an end-user layer.

The first element (sensor network) comprises several nodes integrating different types of sensors. Network nodes are interconnected (using ZigBee protocol), and a gateway is required to receive data from each node and retransmit the information to the cloud system. The gateway must also guarantee the reception of packets from networks nodes and to the cloud system. Furthermore, the cloud system is in charge of receiving the data from the sensor network and providing specific services for data storage, classification, and request. Finally, the last component deals with end-user software tools that provide services for requesting data to the cloud system.

Throughout this section, the different parts of the system are described in detail. First of all, the designed nodes are presented, which are responsible for wirelessly measuring and transmitting the data. This data is received by a gateway, which is the central node of the network. In turn, it allows the user to manage the network, preprocess the data, and connect to the cloud. In the second subsection, the network architecture and operation of the gateway are explained. In addition, the complete process that is required and used in the data-processing tasks is described. Finally, the third subsection describes the services provided by the cloud system for data storing and classifying, and the end-user software tools.

### 2.1. Description of Sensor Nodes

Developed sensor nodes are low-cost and small-sized devices with the ability to collect and transmit information regarding gases in the environment. For this purpose, up to four MOS gas sensors can be connected to each single node. Besides, in order to perform the wireless communication of the nodes, ZigBee technology has been implemented. This is a model that defines a set of communication protocols (which overlaps the IEEE.802.15.4 specification) for low-cost and low-data rate wireless networks. In this design, the devices used for this purpose are the XBee modules from Maxstream. They are radio frequency modules that create ZigBee networks using the IEEE 802.15.4 communication protocol and working in the 2.4-GHz band. They also incorporate a microcontroller that adds a small computing capacity and customization.

The main features of the sensor nodes designed in this work are mainly the following:

- Ability to connect a solar panel to achieve greater autonomy in field applications (7.59 h to months).
- Setting up the heating power of the sensors through the XBee module.
- Controllability of the pump power and electrovalve state for laboratory applications.
- Ability to use any other type of resistive sensor.
- Low dimensions (60 × 40 mm).
- Low current consumption (104 to 270 mA) at low voltages.
- Low cost (<~100 €).

**Figure 2:**
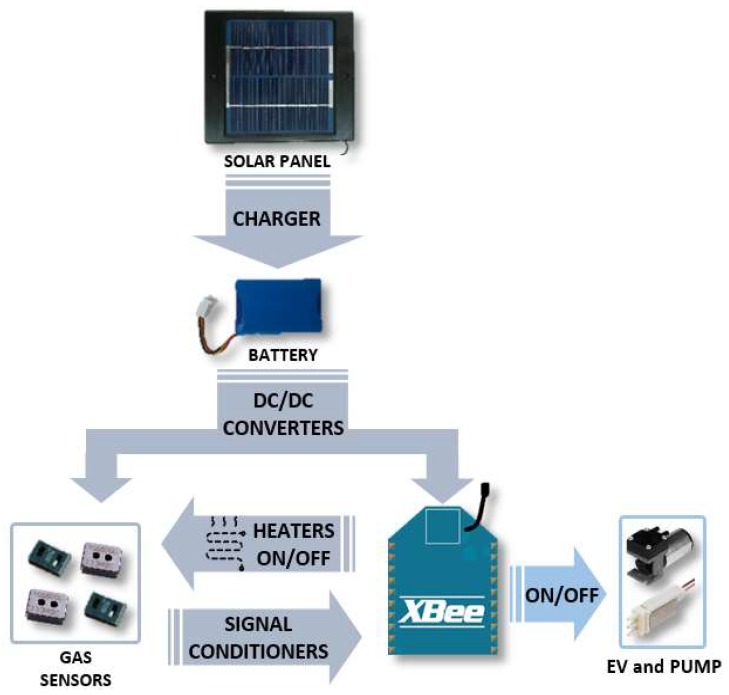
Block diagram of the sensor node.

The block diagram of the module is depicted in Figure 2. Through a battery charge management controller, a solar panel can power the nodes in order to get more autonomy. In turn, a 2050-mAh battery powers the XBee radio frequency module and the gas sensors using DC-DC converters of 3.3 V and 5 V. Operational amplifiers and low-pass filters are used to condition the signals from the gas sensors. These signals are then transmitted to the XBee module, which is responsible for the wireless communication. In addition, it also controls the power of the sensor heaters by using a PWM (pulse width modulation) signal, and disables them when they are not used in order to reduce consumption. Finally, with the purpose of performing measurements under controlled conditions in the laboratory, a pneumatic pump and an electrovalve (EV) can be connected. Its connection/disconnection and power supply are managed by the XBee module as well.

**Figure 3:**
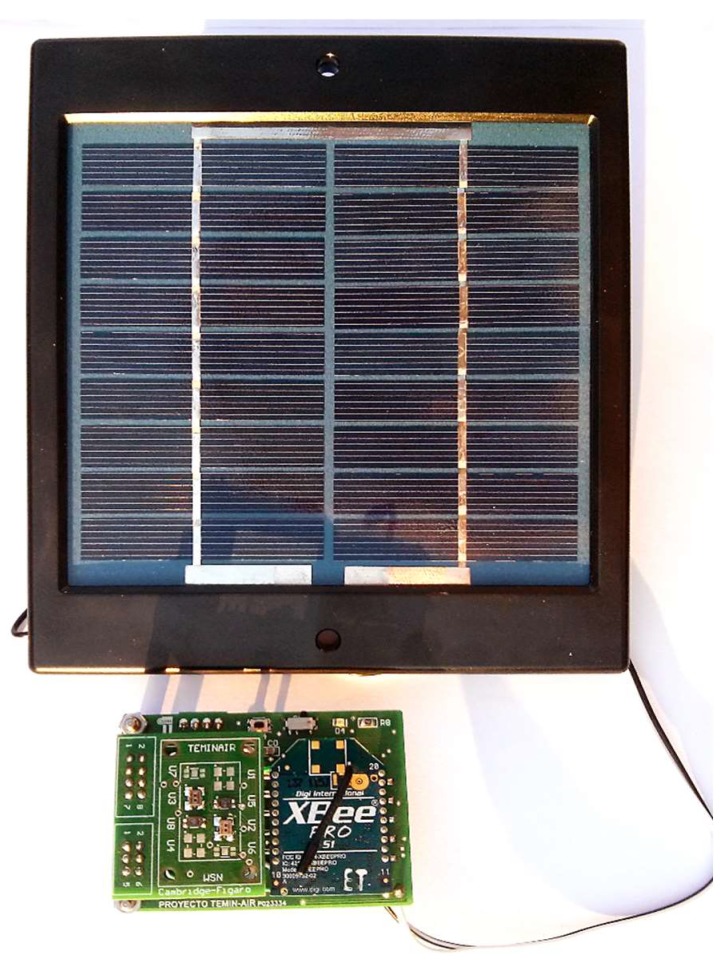
Designed sensor node powered by a solar panel.

Nodes are optimized to support the integration of different gas sensors. The main board enables adding two resistive sensors with the TO-5 package. However, it has a connector that allows plugging in different custom boards containing up to four sensors. For the results presented in this paper, the array is composed by four MOS gas sensors: CCS801 and CCS803 (ams, UK), and two TGS8100 sensors (Figaro, Japan). Figure 3 shows a picture of the real designed node powered by a solar panel.

### 2.2. Description of Gateway Operation and Data Processing

As explained above, each node contains an XBee module in order to wirelessly transmit data relating to the sensors. These nodes are configured with different 16-bit addresses; they are assigned the same PAN (personal area network) identifier and channel, and they are configured as terminal devices. In addition, the required inputs and outputs of the module are configured, and the periodic sending of the data to the coordinator node is programmed.

The information collected by the sensor nodes is sent to the “ConnectPort X4” gateway of Digi (Hopkins, MN, USA) in order to connect the ZigBee network to the cloud system via an Ethernet connection. For this purpose, it has an integrated ZigBee/802.15.4 module. Besides, for its programming, the gateway incorporates a Python® (Beaverton, OR, USA) engine to develop custom applications.

In this study, for laboratory measurements, a Python application has been developed for controlling the sensor network. This application separates and preprocesses the information received by the nodes and sends it to the cloud. In addition, it makes control of the pump and the electrovalve for switching between a reference gas (clean air) and the target gas.

**Figure 4:**
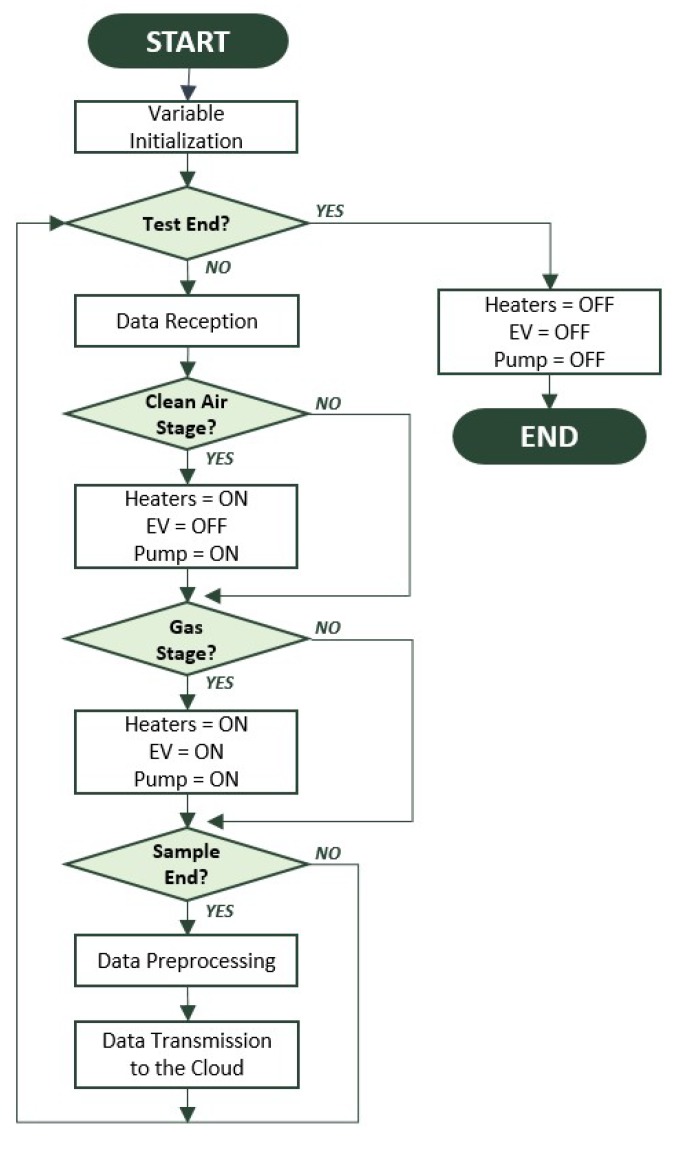
Flow diagram of the measurement program.

A flowchart of the controlled measurement program is shown in Figure 4. In it, after initializing the variables, whether the experiment has come to an end is checked, i.e. whether the desired number of measurement cycles has been carried out. Each cycle consists of the reference gas measurement and the target gas measurement phases. If the experiment has not been completed, the data is received from the sensor nodes in the network. Then, depending on the phase, the analysis stops, and the states of the pump, the electrovalve (EV), and the heating resistances of the sensors (heaters) are modified. That is, in the reference gas measurement phase, the electrovalve is switched off, the pump operates at the determined power, and the sensor heaters are also activated at the determined power. Otherwise, in the target gas measurement phase, the electrovalve is activated, and the pump and the sensors heaters are maintained at the corresponding powers.

When each measurement cycle is completed, the preprocessing stage is carried out. In this stage, the main features from each sensor are extracted and then transmitted to the cloud. The information about each of the four sensors integrated into the device and the corresponding node identification number are received by the cloud system. Finally, when the desired number of cycles is reached, the analysis ends, and the pump, electrovalve, and heating resistor are disconnected.

Otherwise, since the purpose of this device is the measurement of contaminants in the environment, an operation mode of the sensor network in which samples are periodically collected and sent to the data cloud has also been developed. Nodes remain in low-power state during the time intervals between measurements.

**Figure 5:**
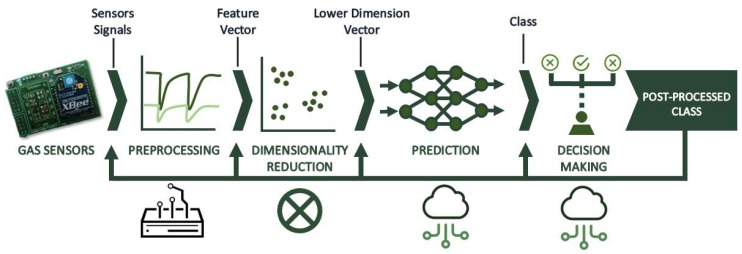
Data processing block diagram.

As explained above, the implementation of pattern recognition and artificial intelligence techniques is required in order to perform contaminant detection tasks. As shown in Figure 5, this process can be divided into four stages [43]: feature extraction and signal preprocessing, dimensionality reduction, prediction, and decision making. In addition, the initial block in this figure represents the whole multisensor system, whose output are the temporary measurements made on several samples. For the present system, the preprocessing stage is implemented at the gateway, dimensionality reduction is not required (although it is included in the results section for better visualization of the data), and the prediction and decision-making stages are performed in the data cloud.

The first stage is the preprocessing of the signal, where the descriptive parameters are extracted from the temporal response of the sensors. From them, the characteristics vector is prepared for further processing. For this purpose, the baseline manipulation technique has been used. This transforms the response of the sensors using their baseline as a reference, i.e. the response of the sensors to the reference gas. In this case, this stage is performed in real time on the gateway by using the relative resistance algorithm (RR) [43].

In the dimensionality reduction stage, the characteristics vector obtained in the previous stage is projected onto a smaller dimensional space to avoid problems associated with large datasets. Principal component analysis (PCA) is one of the most used techniques in this stage. PCA is a powerful, unsupervised, linear pattern recognition technique based on the expansion of Karhunen–Loeve [49], which provided qualitative results on gaseous compounds. This phase has not been implemented in the cloud, because the number of sensors is not high. However, the use of this technique allows the visualization of the data structure by means of graphs. That is why it is used in the results presented in this article, obtaining interpretations that allow a deeper understanding, since it allows analyzing several variables simultaneously.

The resulting low-dimension vector is used to solve a given prediction problem, which is typically clustering, regression, or classification. Regression and classification techniques have been used in this work. In regression tasks, the objective is to predict a set of properties (concentration) of an analyte. For this purpose, the support vector regression (SVR) technique has been selected, as it is generally the most used and recommended in gas sensor applications in the latest literature [50,51,52]. It consists of the use of support vector machine (SVM) in regression tasks [53]. This is done by minimizing the error condition through the so-called "linear ε-insensitive loss function". In addition, the representation by means of kernel functions offers a non-linear problem solution, projecting the information to a space of characteristics of greater dimension that increases the computational capacity of the linear learning machines. With respect to classification tasks, they address the problem of identifying an unknown sample as a class within a learned set. For this, a multilayer perceptron (MLP) [54] with a backpropagation learning algorithm has been trained. An MLP is a feedforward neural network model consisting of multiple layers of nodes in a directed graph. Each layer is fully connected to the next, and each node is a neuron with a non-linear activation function (except the input nodes). Otherwise, the backpropagation algorithm is a learning rule that consists of two stages. First, there is a direct advance stage, in which the external input information on the input nodes is propagated forward to calculate the output information indicators in the output unit. Secondly, there is an inverse phase in which alterations of the connection weights are adjusted based on the differences between the calculated and actual indications in the output units. Classification techniques have been implemented in the cloud system, while regression techniques have been applied externally to study the capacity of the system. It is expected to be implemented in the same way in the cloud sensor network in future works.

The final stage in pattern recognition is the estimation of errors or performance of the trained model using validation techniques. The validation method used in this work is cross-validation, since the same data are used for training and system validation, thus reducing the number of measurements. In this way, the performance of the evaluation is optimized. Specifically, leave-one-out cross-validation (LOOCV) [55] becomes the most appropriate method in this application.

### 2.3. Description of Cloud System and End-User Layer

Wireless sensor networks have the capacity of generating a large volume of data that grows over time, and this is the reason why a high-performance framework must be developed to support system monitoring, performance evaluation, data storage, abnormal situation alerts, end-user services, and even provide processes to obtain new knowledge about the data. The aim of this subsection is to present a cloud framework to monitor and control wireless sensors networks, supplying specific services not only to store the data, but also to apply intelligent mechanisms for data classification and visualization.

The cloud system consists of two different layers: the core and the service layer. The first one integrates those components shared by the services including databases, e-learning algorithms, and connection mechanisms for linking and matching services. The service layer is in charge of publishing different type of services for sensor network monitoring and management. The type of services supported by the cloud system are classified as follows:

- **Storage services**: While data are flooding the cloud from a wireless sensor network, it is mandatory to store the information in a persistent location. To this end, the following storage services have been implemented: a) *create service* provides the necessary actions to create new databases for data storing, b) *connection service* matches a sensor network to a specific database, c) *save service* stores a data sensor network in a concrete database, and d) *retrieve service* requests data from the database and returns the extracted information.
- **End-user services:** These types of services are focused on providing end-user data access. In this sense, the *request identification service* opens a user session to allow data access. Otherwise, the *data visualization service* returns requested data and metadata to present the information graphically. In this proposal, services are not developed independently, since some of them may require other services to provide full functionality. In this regard, the core layer interconnects services to achieve a specific action. For example, the data visualization service is supported by the retrieve service to extract the information from the database.
- **Sensor data services:** These are focused on checking the information retrieved from sensor networks (*check service*). This service also adds new metadata to extend the knowledge of each measurement. For example, when sensor data are received, the service also adds timestamp information and classification values, if they are required.
- **E-learning services:** These support mechanisms classify sensor data through applying e-learning mechanisms (neuronal networks). *Create service* builds a new neuronal network according to several setup values, while the *training service* allows a neuronal network to learn from an initial dataset. Finally, *request service* classifies new sensor network values.
- **Security services:** Security is one of the main problems in cloud-based systems, since services can be available from everywhere. To allow only authorized users to access cloud services, some additional services have been integrated in the proposed cloud system. The *access checking service* verifies if a request is authorized to access the cloud, while the *user management service* allows cloud administrators to manage users and assign privileges.

**Figure 6:**
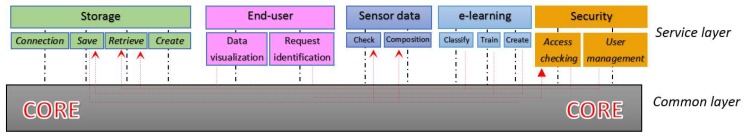
Cloud sensor network framework.

Figure 6 shows a block diagram of the operation of the cloud system.

Furthermore, with respect to services for requesting data from the cloud system, the end-user software tools are configured. The system has been developed with the challenge of being device-independent, which means that users can access the cloud services regardless of the device they use. This goal is achieved by applying Rich Internet Application (RIA) technology in the development process of software applications, which allows accessing cloud services through HTTP protocol only requiring a web browser. RIAs are web applications with extra capabilities to deal with page contents and communicate with the server, and currently, the World Wide Web Consortium (W3C) has published a new recommendation called Accessible Rich Internet Applications (WAI-AIRA) [56] to make accessible web contents. 

To show how the cloud sensor network works, let us consider a sensor network retrieving data from several nodes and sensors. In this situation, the cloud will be requested to process and save the information invoking the *save storage service*, passing all the necessary information to preserve the information into the cloud. At this point, the *security access checking* service is activated to verify access authorization, and next, data coherence is certified by the sensor data check service. Once data is confirmed, the *sensor data composition service* adds new metadata such as timestamps, network identification, source nodes, and even access credentials, in order to supply more information about the measurement. If necessary, data can also be classified at this moment by invoking the e-learning classify service, and this information will be attached to the data for future references. Finally, both the information received and the generated metadata are stored into the cloud by the storage save service. 

**Figure 7:**
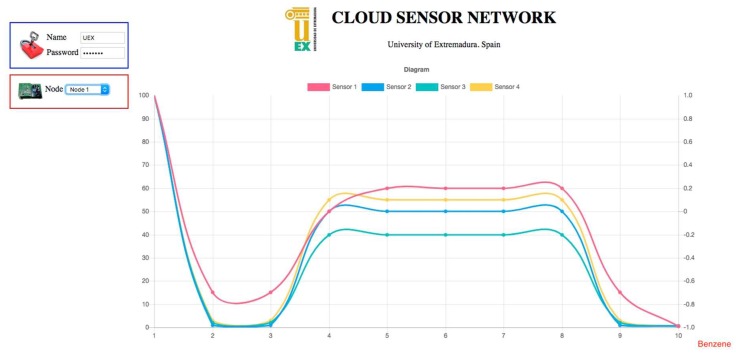
Cloud sensor network caption where benzene is being detected.

In order to help users deal with cloud sensor networks, this section also presents a novel Rich Internet Application (Figure 7). This tool connects to the cloud sensor network, and graphically presents the information requested by the user. The main benefit of asynchronous requests is that clients are not blocked waiting for responses; they can perform parallel tasks until they receive the answers. The basic of this application is requesting information asynchronously from cloud network services, retrieving the information, and presenting it to the user. To perform this task, first, the application requires user credentials and calls the end-user request identification service. Then, the information is requested and classified, if necessary, by invoking the storage retrieve service and the e-learning classify service, respectively. Finally, the end-user data visualization service returns the data and metadata required for presenting the graph.

As it can be seen, the upper left side is divided into two sections: access login and node selection. The first one allows web users to introduce their access information, while the second enables the selection of a specific node from the sensor network. The central area is used for presenting a linear graph reporting on the last measurements taken. This graph presents a different colored line for each sensor, and it is updated every second. The information retrieved is also classified in real-time. For example, Figure 7 shows the classification information provided by the e-learning classification service when the last measurements were taken. In this figure, the x-axis represents the time in seconds, whereas the y-axis shows the preprocessed value detected by the four MOS gas sensors integrated in the device, as described in Section 2.1.

## 3. Discussion and Results

Laboratory measurements of the individual BTEX compounds at different concentrations have been performed to test the operation of the system. First, the conditions and configuration of the measurements are described. Then, the processing procedure and the results obtained are presented. Discrimination and quantification tasks have been accomplished.

### 3.1. Measurement Setup

In this work, the device has been used for the discrimination of BTEX compounds at different concentrations. However, a major advantage of this system is that it could be used for the discrimination of other compounds, thanks to the use of resistive sensors that react to a large number of compounds, and the use of Artificial Neural Networks (ANN), which enable training the system to any application.

**Figure 8:**
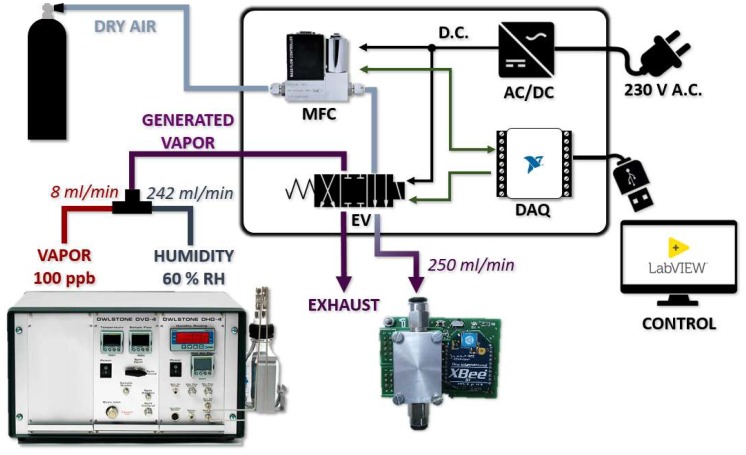
Diagram of the measurement setup architecture.

For this purpose, different compounds have been generated at four different concentrations (10 ppm, 15 ppm, 20 ppm, and 25 ppm) by using permeation tubes, which are polymeric tubes that contain an analyte in solid, liquid, or gaseous state, and are sealed and crimped at both ends. This analyte passes through the walls of the tube at a constant speed under given temperature conditions. It is then mixed and transported by a flow of a carrier diluent gas. Therefore, a one-mL permeation tube has been prepared for each compound. From them, calibrated vapors have been generated using a generating unit (Owlstone OVG-4). This unit also generates a controlled flow of humidity that is added to the original flow. A switch between the reference gas and the target gas has been designed for use with the calibrated vapor generation unit. It consists of two gas inlets (vapor generator and dry air), a mass flow controller responsible for regulating the flow of the reference gas, and an electrovalve whose purpose is to switch the gas circulating in the sensor cell. When the reference gas is passed through the sensor cell, the flow corresponding to the vapor generator, which is continuous, is vented. The operation of the system is controlled through a data acquisition card (with an application developed in LabVIEW), and is powered with 230 V (AC). A diagram of the measuring system is shown in Figure 8.

Ten measurement cycles have been performed for each one of the BTEX compounds and the different concentrations. The cycles have a duration of 10 min: 540 s of dry air flow, and 60 s of pollutant flow.

**Figure 9:**
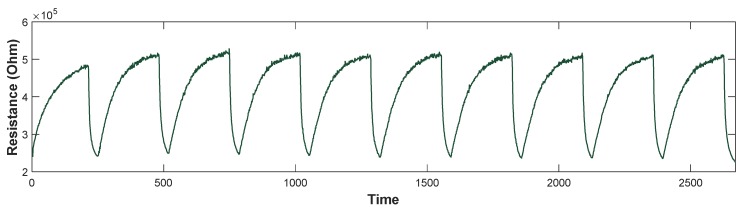
Resistance of sensor three (TGS8100) versus time, corresponding to the benzene measurements at 20 ppb.

As an example, the response of one of the sensors (TGS8100) to the benzene measurements (20 ppb) made is shown in Figure 9.

It can be seen that the first measurement is not entirely correct. This is because the systems were not yet stabilized, and the conditions were not the same. As a result, the first measure has been discarded in all of the cases. Therefore, nine measurements of each compound have been used for processing.

### 3.2. Results

Once the data of the measures performed have been collected, compound discrimination tests of compounds in different concentrations have been carried out. In addition, the possibility of the system to determine the concentration of compounds has also been studied.

#### 3.2.1. Compound Discrimination

In the preprocessing stage, which is performed by the Python program that runs on the gateway, the feature extraction is made. To this end, the RR algorithm is used, which is the ratio between the resistance value of the baseline and the resistance value during exposure to the target compound, as explained above. In this way, it is possible to extract the main information and considerably reduce the size of the data.

**Figure 10:**
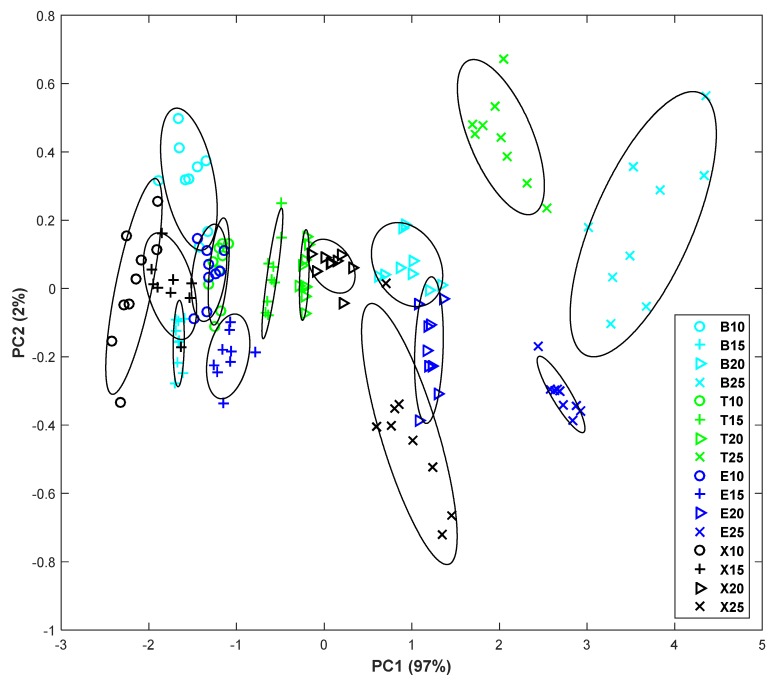
Principal component analysis (PCA) plot of response to the benzene, toluene, ethylbenzene, and xylene (BTEX) compounds. In the legend, the letter identifies the compound, and the number indicates the concentration in ppm.

Then, PCA is carried out to study the resulting distribution of measurements in a graph. The first two principal components for the different concentrations (10 ppm, 15 ppm, 20 ppm, and 25 ppm, respectively) are shown in Figure 10. The different compounds have been represented in different colors to facilitate the reading of the plot: benzene in light blue, toluene in green, ethylbenzene in dark blue, and xylene in black. In the same way, the different concentrations are represented with different symbols. If attention is focused only on groups of equal concentration, it can be noticed that as the concentration increases, the areas are more distinguishable. Thus, in the first case (10 ppb), there is a high overlap in the areas of benzene, toluene, and ethylbenzene. Otherwise, in the graph corresponding to 15 ppb, the overlap is reduced to the benzene and xylene zones while, in the third PCA (20 ppb), there is only a small partial overlap between the ethylbenzene and xylene zones. Finally, in the case of 25 ppb measurements, it can be observed that different areas concerning each compound are clearly separated. By observing the plot in a general way, apart from the overlaps described above, some more appear between compounds of different concentrations: X15 with X10 and E10, T10 with E15, and E20 with X25. However, they are minor overlaps, or only very close approximations. Overall, it suggests that the different areas could be differentiated in most cases.

The results are confirmed by using a neural network classifier (MLP with backpropagation learning algorithm to discriminate among the different compounds. The network architecture consists of three layers: input, hidden, and output. In this case, the input layer consists of a neuron by sensor (four), and the output layer consists of one neuron by compound (16). In the case of the hidden layer, the number of neurons has been optimized, taking into account the success obtained and the time spent on the classification tasks. The optimal number of hidden neurons obtained is 29. LOOCV was used, and the samples were correctly classified into the learned classes. The success rate (proportion of cases correctly classified in the validation as opposed to the total number of cases) obtained is 93.05%. The confusion matrix obtained is presented in Table 1.

#### 3.2.2. Prediction of Compound Concentration

In this case, as before, in the preprocessing stage, the RR baseline manipulation algorithm has been used to extract the feature vector (stage performed in the gateway through the Phyton-designed program). These data have been extracted before being sent to the cloud to study the results in regression tasks.

**Figure 11:**
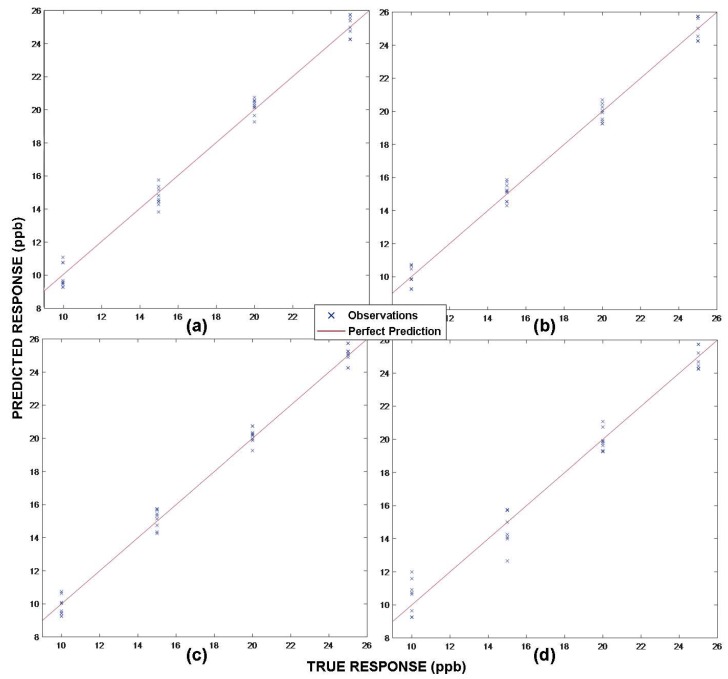
Plot of the predicted concentration results with support vector regression (SVR). (**a**) Benzene; (**b**) Toluene; (**c**) Ethylbenzene; and (**d**) Xylene.

The dimensionality reduction stage is omitted due to the limited size of the data matrix. With respect to the prediction stage, regression tasks are carried out. A Matlab (Mathworks, Natick, MA, USA) toolbox (Statistics and Machine Learning Toolbox™) has been used for this purpose. The SVM technique applied to regression (SVR) is employed. In the implementation of this technique, the kernel function used is a polynomial function of grade three. In Figure 11, the predicted responses versus the true ones are represented for each of the BTEX compounds. It can be noticed that in all of the cases, the maximum errors are around ±1 ppb. The xylene data are those that are farthest from the true response, which can lead to errors between the values referring to concentrations of 10 and 15 ppb.

Table 2 shows some of the main statistical indices of regression performance such as root mean square error (RMSE), coefficient of determination (R-squared), mean squared error (MSE), mean absolute error (MAE), and the time spent on network training.

## 4. Conclusions

A low-cost, low-power, low-size node has been developed for wireless sensor networks for air quality monitoring. These features make it possible to deploy a large number of nodes to create a ubiquitous sensor network. The use of a gateway enables the preprocessing before sending the data, reducing its dimensionality and connecting the nodes directly to the cloud, where the data is stored, processed, and displayed.

The aim of the network is the detection of air pollutants in large areas. Its efficiency has been verified by detecting and quantifying volatile organic compounds (BTEX). Pattern recognition techniques have been used for this purpose. The results indicate a proper performance of the system in both tasks, achieving success rates of discrimination of 93.05% and determination coefficients around 0.99 in the quantification tasks (regression). 

The parallel placement of these systems with traditional VOC monitoring systems could allow the calibration and training of this system in field applications. Future research includes the deployment of a large number of nodes in the monitoring area, real conditions testing, and the field calibration of the sensors. Relating air quality to gas detection is another work in progress that will report important information for people’s health in real time.

## Figures and Tables

**Table 1:** Confusion matrix obtained in leave-one-out cross-validation (LOOCV).

	B10	B15	B20	B25	T10	T15	T20	T25	E10	E15	E20	E25	X10	X15	X20	X25
**B10**	**8**	0	0	0	1	0	0	0	0	0	0	0	0	0	0	0
**B15**	0	**8**	0	0	0	0	0	0	1	0	0	0	0	0	0	0
**B20**	0	0	**9**	0	0	0	0	0	0	0	0	0	0	0	0	0
**B25**	0	0	0	**9**	0	0	0	0	0	0	0	0	0	0	0	0
**T10**	0	0	0	0	**7**	0	0	0	1	1	0	0	0	0	0	0
**T15**	0	0	0	0	0	**9**	0	0	0	0	0	0	0	0	0	0
**T20**	0	0	0	0	0	0	**9**	0	0	0	0	0	0	0	0	0
**T25**	0	0	0	0	0	0	0	**9**	0	0	0	0	0	0	0	0
**E10**	0	1	0	0	1	0	0	0	**7**	0	0	0	0	0	0	0
**E15**	0	0	0	0	0	0	0	0	0	**9**	0	0	0	0	0	0
**E20**	0	0	0	0	0	0	0	0	0	0	**9**	0	0	0	0	0
**E25**	0	0	0	0	0	0	0	0	0	0	0	**9**	0	0	0	0
**X10**	0	0	0	0	0	0	0	0	0	0	0	0	**8**	1	0	0
**X15**	0	0	0	0	0	0	0	0	0	0	0	0	0	**9**	0	0
**X20**	0	0	0	0	0	0	0	0	0	0	0	0	0	0	**9**	0
**X25**	0	0	0	0	0	0	0	0	0	0	3	0	0	0	0	**6**

**Table 2:** Statistical indices of each of the presented regressions (BTEX). RMSE: root mean square error, MSE: mean squared error, MAE: mean absolute error.

	RMSE	R-Squared	MSE	MAE	Training Speed (s)
Benzene	0.6017	0.99	0.3620	0.5428	1.36
Toluene	0.5741	0.99	0.3296	0.5126	0.52
Ethylbenzene	0.5289	0.99	0.2797	0.4548	0.80
Xylene	0.8727	0.98	0.7615	0.7442	1.03
